# Bischoff on Maturation of the Ovum

**Published:** 1848-01

**Authors:** 


*Bischoff on Maturation of the Ovum.*—We learn by the Annalist that a translation of the "Proofs of the Maturation of the ovum *sine coitu*," &c., by Bischoff, Prof. of Comp. Anat. in the University of Berlin, will shortly be published by *the Woods*, of New-York city. The readers of medical journals are already somewhat acquainted with the highly interesting observations of the German Professor, and his work will doubtless be welcome.

